# Trauma team activation for pediatric patients in Denmark: a multicenter study of criteria, organization, and injury severity

**DOI:** 10.1007/s00068-026-03208-2

**Published:** 2026-05-11

**Authors:** Christina Højfeldt Nordestgaard, Martin Faurholdt Gude, Sara Viskum Leth, Nikolaj Raaber

**Affiliations:** 1 Research Center for Emergency Medicine, Department of Clinical Medicine, Aarhus University, Aarhus, Denmark; 2 Department of Research & Development, Prehospital Emergency Medical Services, Central Denmark Region, Aarhus, Denmark; 3 Department of Clinical Medicine, Aarhus University, Aarhus, Denmark; 4 Department of Emergency Medicine, Aarhus University Hospital, Aarhus, Denmark

**Keywords:** Pediatric trauma, Trauma team activation criteria, Triage, Injury Severity Score, Overtriage

## Abstract

**Background:**

Pediatric trauma team activation (TTA) is intended to ensure timely management of severely injured children, yet both prehospital visitation practices and in-hospital TTA criteria vary across trauma systems. The aim of this study was to describe and compare pediatric TTA criteria, organizational models, and injury severity across all Danish level I trauma centers.

**Methods:**

We conducted a retrospective multicenter cohort study including all pediatric trauma patients (< 18 years) admitted via TTA at the four Danish level I trauma centers between 2014 and 2024. Center-specific prehospital and in-hospital TTA protocols were obtained through structured inquiries. Injury severity was assessed using Injury Severity Score (ISS) and Abbreviated Injury Scale (AIS). Overtriage was defined as TTA in patients with ISS < 15.

**Results:**

A total of 3,452 pediatric trauma patients were included. Two distinct TTA models (single-criterion and point-based) and four organizational structures were identified. Overall, 14% of patients had ISS ≥ 15, corresponding to high overtriage across all centers. Overtriage rates ranged from 81% to 95% and were highest at centers using point-based TTA models. Mortality was low and did not differ significantly between centers. Among the most severely injured patients (ISS ≥ 25), head and thoracic injuries predominated.

**Conclusion:**

Pediatric trauma triage in Denmark shows substantial inter-center variation combined with consistently high overtriage. These findings suggest a misalignment between current TTA practices and pediatric injury severity profiles. Greater alignment of pediatric-specific triage criteria across prehospital and in-hospital settings may support more accurate TTA, promote consistent care, and improve resource utilization across trauma centers.

**Supplementary Information:**

The online version contains supplementary material available at 10.1007/s00068-026-03208-2.

## Introduction

Trauma remains the leading cause of mortality among children in industrialized countries and constitutes a substantial burden of hospital admissions within pediatric populations [1, 2]. To reduce trauma-related mortality, emergency departments (EDs) rely on structured triage systems to allocate resources according to injury severity [3, 4]. Hospital trauma team activation (TTA) is a key element of these systems, aiming to rapidly identify critically injured children requiring immediate multidisciplinary resuscitation. Previous studies have demonstrated an association between trauma team involvement and reduced mortality among severely injured pediatric patients, underscoring the importance of timely and appropriate TTA [5]. While dedicated pediatric trauma centers have not consistently shown superior outcomes compared with adult trauma centers after adjustment for case mix and injury severity, treatment at trauma centers verified by the American College of Surgeons has been associated with improved survival, reflecting the importance of standardized trauma system organization and quality requirements [6]. Accurate triage is therefore essential. Undertriage may delay life-saving interventions for severely injured children, whereas overtriage—activation of trauma teams for patients with minor injuries—can lead to inefficient use of resources, increased costs, and emotional distress for children and their families [7, 8].

Despite this, no universally validated or age-adjusted pediatric TTA criteria currently exist [9]. Existing TTA protocols typically combine physiological parameters, anatomical injury patterns, and mechanisms of injury (MOI), yet substantial variation persists both internationally and within individual countries [9–11]. Several pediatric triage models, including point-based and single-criterion approaches, have been proposed, but evidence regarding their diagnostic performance and impact on clinical outcomes remains limited and inconsistent [12, 13]. Although pediatric trauma epidemiology, MOI, and outcomes have been described in multiple cohorts [14–18], the consequences of inter-center variation in TTA criteria for pediatric patient outcomes have not been systematically evaluated in Denmark.

This study aimed to evaluate pediatric TTA protocols across all four Danish level I trauma centers, focusing on variation in activation criteria, organizational structure, and associated patient outcomes. By providing a national overview of current practice, the study seeks to inform future development of standardized, pediatric-specific trauma triage guidelines in comparable healthcare systems.

## Methods

### Study design

This retrospective, multicenter cohort study included all pediatric trauma patients (< 18 years) admitted via TTA at the four Danish level I trauma centers between January 2014 and December 2024. A closed-cohort design was used, combining registry-based data from the Danish Trauma Registry with structured survey information on TTA protocols and organizational models obtained from each center. The study population was restricted to level I trauma centers, as Danish Trauma Registry only includes patients with TTA at these centers, and comparable data from regional hospitals were not available for the study period. The study was conducted and reported in accordance with the STROBE guidelines (Appendix 1 - S1) [19].

### Setting

During the study period, Denmark was divided into five geographical regions, with four level I trauma centers located at Aalborg University Hospital, Aarhus University Hospital, Odense University Hospital, and Copenhagen University Hospital. These centers provide comprehensive multidisciplinary trauma care and collectively manage all severely injured children requiring specialized trauma treatment. Denmark has a population of approximately 6 million, of whom about 20% are under 18 years of age [20].

The prehospital emergency medical service (EMS) comprises ground and air ambulance services. Prehospital personnel notify the receiving ED in advance to support hospital triage decisions. Children with minor injuries are typically referred to regional hospitals with standard ED and trauma facilities, whereas children with severe injuries are referred directly to or secondarily transferred to a level I trauma center, where specialized pediatric trauma resources are available. These centers also receive all types of trauma patients from their own catchment areas. As some severely injured children are initially managed at regional hospitals before transfer, interhospital transfer is an integral component of the system. Information on prehospital visitation criteria was requested from the ambulance service organizations in each of the five Danish regions (Appendix 2 - Table S1-S2).

Across Denmark, the four level I trauma centers use different TTA protocols, resulting in variation in activation thresholds that may not accurately reflect injury severity [21]. For the purposes of this study, the five regions and four trauma centers were anonymized and designated as regions 1–5 and Trauma Centers 1–4 (TC1-TC4) to facilitate comparative analyses across centers.

### Study population

Eligible participants were pediatric trauma patients aged 0–17 years with an Injury Severity Score (ISS) > 0 who were received by a multidisciplinary trauma team at one of the four Danish level I trauma centers between January 1, 2014, and December 31, 2024.

### Data sources and survey data

Patient-level data were retrieved from the Danish Trauma Registry, a national registry established in 2014 to monitor and improve the quality of trauma care at Danish trauma centers. Extracted variables included age, sex, transfer status (direct or secondary transfer), ISS, 30-day mortality, Abbreviated Injury Scale (AIS), prehospital systolic blood pressure (SBP), and prehospital Glasgow Coma Scale (GCS) score.

### Exposure

TTA protocols were used to characterize each trauma center’s approach to pediatric trauma care, with each of the four level I trauma centers representing a distinct center-specific exposure (TC1-TC4). Information on current prehospital and in-hospital TTA protocols was obtained through structured inquiries to trauma care physicians at each center (Appendix 1 - S2).

Prehospital visitation criteria for pediatric trauma differed across regions. In some regions, transport decisions relied primarily on clinical judgment by prehospital physicians, whereas others applied predefined criteria, including ABCDE-based triggers for direct transfer to a level I trauma center (Appendix 2 - Tables S1-S2).

All trauma centers had formalized TTA protocols, but organization and activation criteria varied. TTA could be initiated by prehospital physicians, emergency department staff, nurse-led protocols, or, at one center, directly by ambulance personnel. Final activation confirmation was handled by physicians, nurses, or centralized coordination systems (Appendix 2 - Table S3).

Across centers, TTA criteria incorporated anatomical findings, physiological parameters, and MOI, but differed in structure. Two main models were identified: a single-criterion model, in which any qualifying anatomical, physiological, or mechanism-based criterion triggered activation, and a point-based model, requiring accumulation of multiple criteria points (Appendix 2 - Table S4). Graded versus absolute thresholds (e.g., Glasgow Coma Scale cut-offs) varied accordingly. None of the centers applied pediatric-specific TTA criteria; pediatric patients were assessed using adult criteria with limited age-related adjustments.

Pediatric trauma team size ranged from 12 to 16 members across centers (Appendix 2 - Table S3).

### Outcomes

The primary outcome was overtriage, defined as TTA in patients with an ISS < 15. Secondary outcomes included injury distribution by AIS and mortality at 3 and 30 days.

Injury severity was assessed using AIS and ISS as part of routine Danish trauma registry practice. AIS classifies injuries by anatomical region and severity on a six-point scale from minor to lethal and was assigned by certified healthcare professionals in accordance with national standards. ISS was derived from AIS as the sum of the squares of the three highest scores from different body regions, and an ISS ≥ 15 was used to define severe injury [22, 23]. AIS coding was not available for all patients during the study period, and the registry does not allow differentiation between missing AIS data and true absence of injury. Consequently, ISS could not be calculated for all patients.

Overtriage was calculated for each trauma center as the proportion of patients receiving TTA despite having ISS < 15 and was evaluated against reference thresholds proposed by the American College of Surgeons Committee on Trauma, which consider overtriage rates of 25–50% acceptable in trauma systems [24].

### Statistical analysis

Descriptive statistics were used to summarize the data. Categorical variables are presented as counts and percentages, normally distributed continuous variables as means with standard deviations, and non-normally distributed variables as medians with interquartile ranges. Differences in overtriage rates across trauma centers (TC1-TC4) were examined using linear regression with robust variance estimation, defining overtriage as TTA in patients with an ISS < 15.

Mortality outcomes were derived from Danish Trauma Registry data, and 3-day and 30-day mortality proportions were calculated based on recorded dates of admission and death. Patients could contribute more than one observation if they experienced multiple TTAs during the study period; each activation was treated as a separate event if occurring at least 30 days apart.

Adjusted analyses were performed using inverse probability of treatment weighting to address covariate imbalance. Overtriage models were adjusted for age and sex, and mortality models were additionally adjusted for ISS. All analyses were conducted using Stata version 18.5 (StataCorp LLC, College Station, TX, USA).

### Ethics approval and consent to participate

The study was approved by the Legal Department of the Central Denmark Region (journal no. 1-16-02-89-25), with a formal waiver of patient consent. All data were stored in accordance with the Central Denmark Region data protection guidelines using a secure, regionally governed research data platform.

## Results

A total of 3,452 children and adolescents aged 0–17 years were included between 2014 and 2024 following TTA. Case volume varied substantially across the four trauma centers. Trauma Center 2 (TC2) received the largest number of pediatric trauma patients, with 1,280 cases (37.1%; 95% CI 35.5–38.7), followed by TC4 with 987 cases (28.6%; 95% CI 27.1–30.1), TC1 with 681 cases (19.7%; 95% CI 18.4–21.1), and TC3 with 504 cases (14.6%; 95% CI 13.5–15.8) (Table 1).

The overall median age was 12 years (IQR 5–16), and boys accounted for 2,124 cases (61.5%; 95% CI 59.9–63.1). Injury severity data were available for 1,507 patients (43.7%; 95% CI 42.0–45.3).

ISS completeness varied across trauma centers, ranging from 33.7% (95% CI 30.9–36.7) at TC4 to 52.3% (95% CI 49.6–55.1) at TC2, with intermediate proportions at TC3 (43.3%; 95% CI 39.0–47.6) and TC1 (42.0%; 95% CI 38.3–45.7). Patients with and without available ISS were similar in median age (11.7 years [IQR 4–15] and 11.7 years [IQR 5–15]), transfer rates (14.3%; 95% CI 12.6–16.1 and 11.1%; 95% CI 9.7–12.5), and 30-day mortality (1.7%; 95% CI 1.2–2.5 and 2.5%; 95% CI 1.9–3.3).

**Table 1:** Patient characteristics, interhospital transfers, injury severity, and outcomes by trauma center

	TC1 *n* (%, 95% CI)	TC2 *n* (%, 95% CI)	TC3 *n* (%,95% CI)	TC4 *n* (%,95% CI)	All Trauma centers *n* (%,95% CI)
Patient cases, *n* (%)	681 (20)	1280 (37)	504 (15)	987 (28)	3452
Age, median (IQR)	12 (5 to 16)	11 (4 to 16)	14 (8 to 17)	11 (5 to 15)	12 (5 to 16)
Sex (boys), n (%)	405 (60)	813 (64)	311 (62)	595 (60)	2124 (62)
Transfers, n (%)	79 (12)	235 (18)	12 (2)	104 (11)	430 (13)
Injury severity score (ISS)					
Patients with non-missing ISS, n (%)	286 (42)	670 (52)	218 (43)	333 (34)	1507 (44)
ISS, median (IQR)	8 (2 to 13)	5 (2 to 12)	4 (1 to 9)	1 (1 to 4)	4 (1 to 10)
ISS ≥ 15, n (%)	54 (19)	123 (18)	24 (11)	16 (5)	217 (14)
ISS ≥ 25, n (%)	24 (8)	34 (5)	8 (4)	0 (0)	66 (6)

Transfers refer to interhospital transport from another hospital to a level I trauma center. Percentages are calculated based on non-missing data. ISS = Injury severity score; IQR = Interquartile rate; TC = Trauma center; n = number

### Injury severity

Injury severity varied markedly between trauma centers (Table 1). Median ISS ranged from 1 (IQR 1–4) at TC4 to 8 (IQR 2–13) at TC1. Among patients with available ISS data, 217 (14.4%; 95% CI 12.7–16.3) had ISS ≥ 15, and 66 (6.1%; 95% CI 5.0–7.4) had ISS ≥ 25.

The proportion of TTA associated with ISS ≥ 15 differed substantially across centers. This proportion was highest at TC1 (18.9%; 95% CI 14.7–23.9) and TC2 (18.4%; 95% CI 15.6–21.5), lower at TC3 (11.0%; 95% CI 7.5–15.9), and lowest at TC4 (4.8%; 95% CI 3.0–7.7). Overall, 1,300 of 1,507 patients (85.6%; 95% CI 83.7–87.3) had ISS < 15, indicating a high degree of overtriage.

### Transfers and age-related patterns

**Fig. 1:**
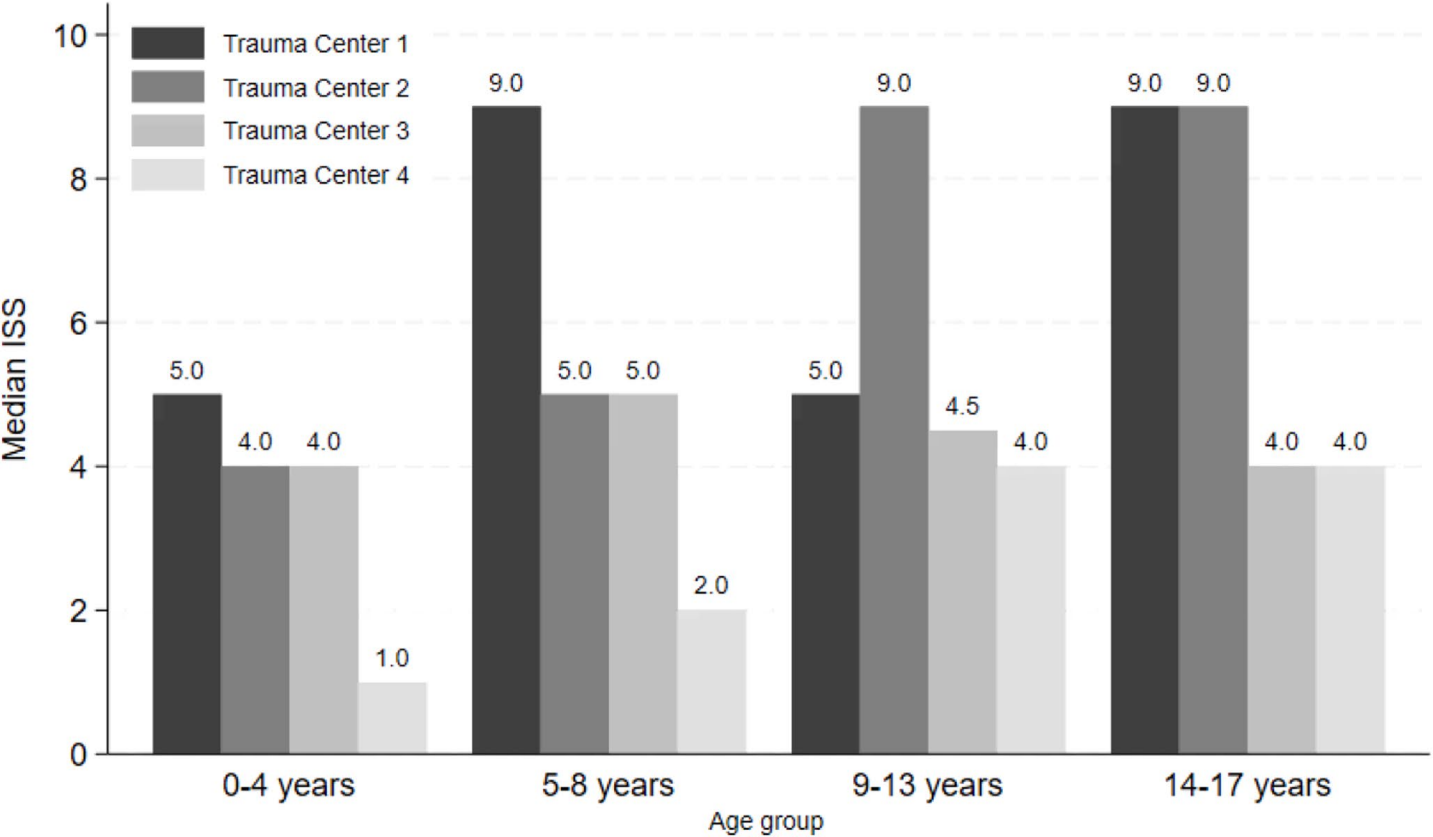
Median injury severity score by age group and trauma center. ISS = Injury Severity Score

Interhospital transfer rates were lowest at TC3 (2%) compared with the other trauma centers (11–18%) (Table 1). Across all trauma centers, injury severity increased with age. The youngest children (0–4 years) consistently demonstrated the lowest median ISS, whereas higher median ISS values were observed in older age groups, as illustrated in Fig. 1. Overall, most pediatric trauma patients sustained minor injuries, with 925 children (61%) classified as ISS 1–8. Injury severity increased with age: the median age was 10 years (IQR 4–15) among patients with minor injuries and 14 years (IQR 9–17) among those with very severe injuries (ISS ≥ 25) (Fig. 1; Table 2).

### Physiological parameters

Analysis across age and injury severity groups revealed systematic differences in physiological parameters (Table 2). Prehospital systolic blood pressure decreased with increasing ISS, with younger children consistently presenting with lower values than older age groups. Similarly GCS scores declined with increasing injury severity, most notably among adolescents.

**Table 2:** Characteristics of trauma from mild to severe Injury by region and age

ISS	ISS 1–8 (minor)	ISS 9–15 (moderate)	ISS 16–24 (severe)	ISS ≥ 25 (Very severe)	Total
Patient cases, N (%)	925 (61)	365 (24)	125 (9)	92 (6)	1507 (44)
Age, median (IQR)	10 (4 to 15)	12 (5 to 16)	13 (7 to 16)	14 (9 to 17)	12 (5 to 16)
ISS, median (IQR)	2 (1 to 4)	10 (9 to 12)	17 (16 to 19)	29 (25 to 38)	4 (1 to 10)
Polytrauma, N (%)	0 (0)	0 (0)	40 (32)	55 (60)	95 (6)
30-days mortality, N (%, 95% CI)	0 (0, 0–0.004)	2 (0.55, 0.001–0.019)	6 (4.8, 0.018–0.102)	18 (19.57, 0.120–0.291)	26 (1.73,0.011–0.025)
Vital signs by age-group and ISS					
0–4 years					
N of patients (%)	294 (70)	86 (20)	23 (6)	17 (4)	420 (28)
SBP, mean (SD)	105 (22)	103 (20)	110 (36)	87 (19)	105 (23)
GCS, median (IQR)	15 (14 to 15)	15 (14 to 15)	15 (11 to 15)	13 (12 to 14)	15 (14 to 15)
5–8 years					
N of patients (%)	133 (64)	55 (26)	15 (7)	7 (3)	210 (14)
SBP, mean (SD)	110 (20)	116 (17)	100 (13)	98 (0)	112 (19)
GCS, median (IQR)	15 (14 to 15)	15 (15 to 15)	15 (15 to 15)	15 (15 to 15)	15 (14 to 15)
9–13 years					
N of patients (%)	172 (59)	73 (25)	28 (10)	19 (6)	292 (19)
SBP, mean (SD)	115 (18)	119 (18)	103 (29)	95 (42)	114 (22)
GCS, median (IQR)	15 (14 to 15)	15 (14 to 15)	15 (14 to 15)	12 (4 to 15)	15 (14 to 15)
14–17 years					
N of patients (%)	326 (56)	151 (26)	59 (10)	49 (8)	585 (39)
SBP, mean (SD)	123 (21)	123 (23)	122 (26)	96 (40)	121 (25)
GCS, median (IQR)	15 (14 to 15)	15 (14 to 15)	15 (14 to 15)	9 (3 to 15)	15 (14 to 15)

N = number; ISS = Injury Severity Score; IQR = Interquartile rate; SD = standard deviation; Polytrauma = 2 injuries with AIS ≥ 3; SBP = Systolic blood pressure, prehospital; GCS = Glassgow coma scale, prehospital. Missing data were excluded from calculations

### Overtriage and mortality

Overtriage rates were high across all trauma centers, ranging from 81.1% (95% CI 76.6–85.7) at TC1 to 95.2% (95% CI 92.9–97.5) at TC4 (Table 3). Using TC1 as the reference, regression analyses demonstrated increasing absolute differences in overtriage at TC3 and TC4, both before and after adjustment. Overtriage rates remained relatively stable across time intervals within all trauma centers, with minor fluctuations and no consistent temporal trend (Appendix 2 - Table S5). Due to limited availability of ISS data in the earliest study period (2014–2016), temporal analyses were restricted to later intervals.

Overall mortality was low. Among 3,016 patients with available mortality data, 64 deaths occurred within 30 days, corresponding to a 30-day mortality rate of 2.2% (95% CI 1.7–2.7). Most deaths (83%) occurred within the first three days after trauma. Mortality increased markedly with injury severity, from 0% in patients with minor injuries to approximately 20% among those with ISS ≥ 25 (Table 2).

**Fig. 2:**
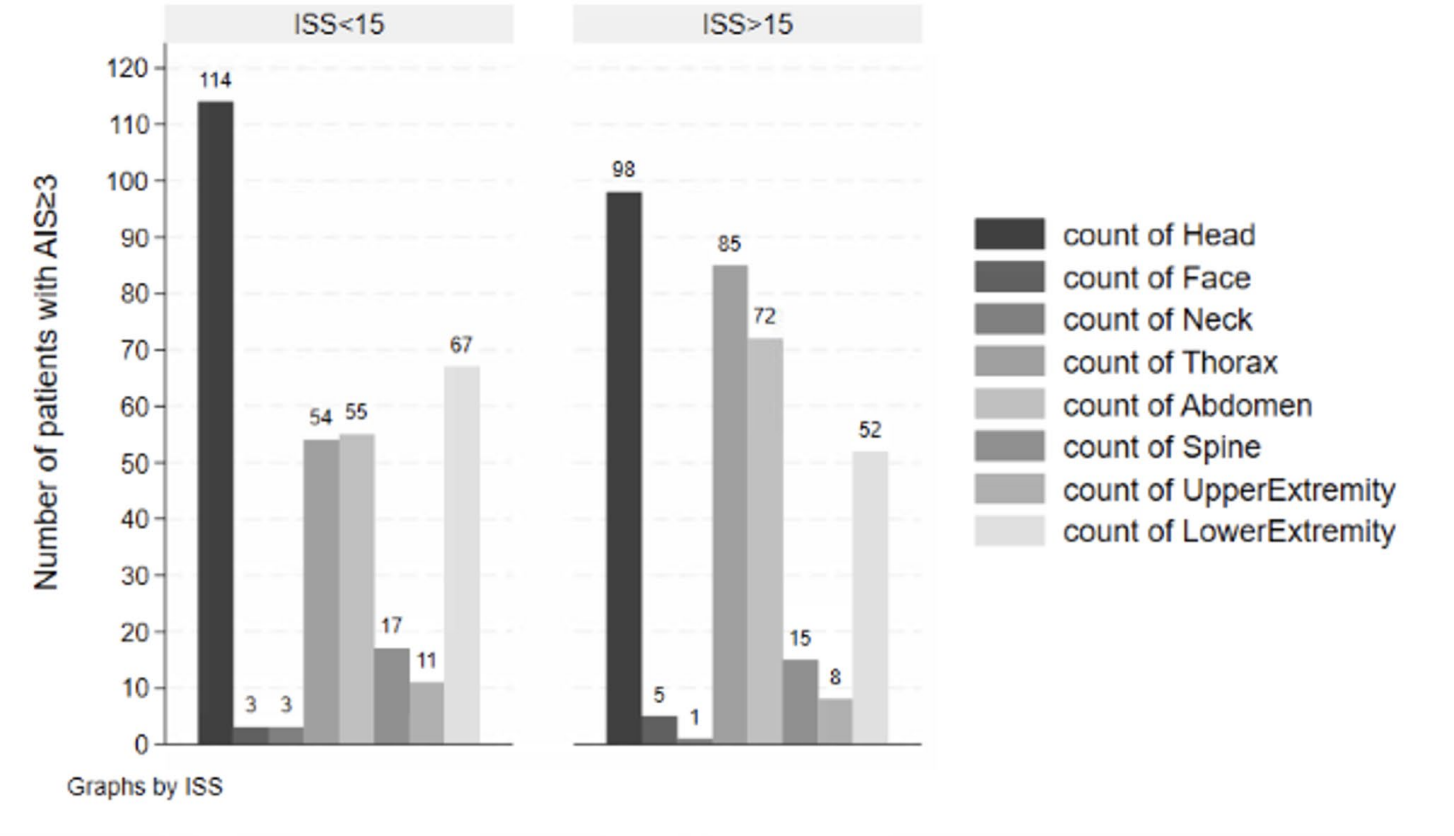
Proportion of patients with isolated injuries by body region and ISS (1–15 vs. 16–75). AIS = Abbreviated Injury Scale; ISS = Injury Severity Score

Across all injury severity categories, head injuries were the most frequent. Thoracic and abdominal injuries became increasingly common with higher ISS, as shown in Fig. 2 and Appendix 2 (Table S6).

**Table 3:** Overtriage rates at the level I trauma centers in Denmark

Triage	TC1, %, 95% CI	TC2, %, 95% CI	TC3, %,95% CI	TC4, %,95% CI
Overtriage rates (ISS < 15), % (CI)	81.1 (76.6 to 85.7)	81.6 (78.7 to 84.6)	89.0 (84.8 to 93.2)	95.2 (92.9 to 97.5)
Overtriage absolute difference, pp (CI)	Ref	0.5 (-0.5 to + 0.6) (*N* = 956)	7.9 (1.7 to 14.1) (*N* = 504)	14.1 (9.0 to 19.2) (*N* = 619)
Overtriage absolute difference, adjusted*, pp (CI)	Ref	0.1 (-5.3 to + 5.5) (*N* = 956)	8.9 (2.7 to 15.2) (*N* = 504)	13.8 (8.7–18.9) (*N* = 619)
Mortality 30-day				
30 day-Mortality, % (CI)	2.7 (1.4 to 4.0)	2.4 (1.5 to 3.3)	1.4 (0.3 to 2.6)	1.7 (0.8 to 2.6)
30-day mortality absolute difference, % (CI)	Ref	-0.3 (-1.9 to + 1.3) (*N* = 1,716)	-1.3 (-3.0 to + 0.5) (*N* = 1,008)	-1.0 (-2.6 to + 0.6) (*N* = 1,472)
30-day absolute difference, adjusted**, % (CI)	Ref	-0.2 (-2.1 to + 1.7) (*N* = 837)	6.2 (-7.1 to + 19.4) (*N* = 435)	5.0 (-1.2 to + 11.1) (*N* = 543)

ISS = Injury Severity Score; TC = Trauma Center

*Adjusted for age and sex; **additionally adjusted for ISS; Numbers (N) in analyses in parentheses

## Discussion

This nationwide cohort study is, to our knowledge, the first to compare pediatric TTA practices and outcomes across all four designated Danish level I trauma centers. Using uniform registry data combined with center-specific protocol information, we identified substantial variation in TTA criteria and organization, accompanied by consistently high overtriage rates. While previous Danish studies have described local pediatric trauma epidemiology and outcomes [25], our study provides a national, comparative perspective that may inform trauma system organization.

### Variation in triage and overtriage

A central finding of this study was the marked variation in pediatric trauma triage across Danish level I trauma centers, particularly in prehospital visitation practices and in-hospital TTA criteria. Across all centers, trauma teams were frequently activated for children with an ISS < 15, indicating a substantial degree of overtriage. However, the magnitude of overtriage varied between centers, suggesting that differences in organizational structure and triage models influence activation thresholds.

Prehospital visitation practices differed considerably between regions, ranging from reliance on clinical judgment to predefined visitation criteria. Notably, the trauma center serving the region without formal prehospital visitation criteria did not exhibit the highest overtriage rate, indicating that experienced clinical judgment may perform adequately in some settings. Nevertheless, the absence of standardized criteria may increase the risk of undertriage. Previous studies indicate that strict adherence to prehospital protocols improves the identification of severely injured patients [26]. Undertriage could not be directly assessed in this study due to lack of data on patients not initially received as TTA; however, secondary interhospital transfers suggest that some children initially managed at regional hospitals later required higher-level trauma care. In this context, secondary interhospital transfer rates were lowest at centers with the highest overtriage (2–11%) and higher at centers with lower overtriage (12–18%), illustrating that observed overtriage rates arise from the interaction between prehospital routing decisions and in-hospital activation thresholds rather than from either component alone. Their transfers may reflect either unrecognized severe injuries or the need for specialized services–such as pediatric or neurosurgical care–not available at the referring hospital.

### TTA criteria

We identified notable discrepancies in pediatric TTA criteria, particularly regarding the role of MOI. At TC1 and TC2, MOI functions as an independent trigger for TTA, whereas at TC3 and TC4, MOI contributes only as part of a point-based scoring system requiring additional criteria for activation. A recent study identified MOI as a strong driver of overtriage in pediatric trauma [27], suggesting that higher overtriage might be expected when MOI is used as a single activation criterion. However, this pattern was not observed in our data, as TC1 and TC2 had the lowest proportions of patients with ISS < 15. Interpretation is limited by lack of case-level MOI data, and future studies should systematically capture individual TTA criteria to better evaluate the contribution of MOI to triage accuracy.

### Injury severity

Median ISS and the proportion of patients with ISS ≥ 15 varied across centers, but overall, only 14% of pediatric patients met this threshold, indicating that most TTAs involved children with relatively minor injuries. Consequently, 86% of activations occurred in patients with ISS < 15, exceeding the overtriage range of 25–50% recommended by the American College of Surgeons Committee on Trauma. Overtriage was highest at TC3 (89.0%) and TC4 (95.2%), both using point-based TTA models, whereas TC1 (81.1%) and TC2 (81.6%), using single-criterion models, had lower overtriage rates. This pattern suggests that differences in TTA structure may be associated with differences in activation thresholds. High levels of pediatric overtriage have also been reported in previous Danish studies [15, 25], and in international cohorts from Switzerland and the United States [28, 29], indicating that pediatric overtriage remains a persistent challenge across trauma systems.

Applying a more stringent definition of severe injury (ISS ≥ 25), which may better reflect clinically significant pediatric trauma [30], would further increase the estimated degree of overtriage, as 96% of patients in our total cohort had ISS < 25. While some degree of overtriage may be unavoidable in pediatric trauma care, excessive activation has important organizational consequences, including inefficient resource utilization and increased psychological burden for children and families [8].

### Mortality

Overall mortality was low. Although unadjusted analyses suggested lower mortality at centers with higher overtriage, this pattern reversed after adjustment for ISS, age, and sex. Differences were small, not statistically significant, and based on few events, and should therefore be interpreted cautiously. Collectively, these findings suggest that variation in TTA criteria primarily influences organizational outcomes such as overtriage rather than mortality, which remained consistently low across centers.

### Injury by body region

ISS does not fully capture physiological compromise or the cumulative impact of multiple injuries within a single anatomical region. Injuries to the head and thorax, which are associated with high pediatric mortality [31, 32], may therefore carry greater clinical relevance than higher composite ISS values driven by extremity injuries. In our cohort, head injury was the most frequent and severe injury among patients with ISS ≥ 25 (44%), underscoring its central role in pediatric trauma severity [30]. All TTA protocols incorporated head injury indicators, primarily through GCS thresholds, whereas extremity injuries contributed little to severe injury classification (8–11% of ISS ≥ 15). This finding suggests that only the most severe fractures warrant consideration as independent TTA triggers. However, the present study was not designed to assess the independent impact of specific injury patterns on outcomes or TTA criteria.

### Age-related trends

Injury severity increased with age, with adolescents exhibiting the highest ISS across centers, consistent with prior Danish and international studies [15, 25, 33]. Greater exposure to high-energy mechanisms likely contributes to this pattern [33, 34]. In line with this age-related gradient, the youngest children (0–4 years) had the lowest median ISS across all centers. Despite lower injury severity, young children may still trigger TTA due to a more cautious triage approach, reflecting challenges in clinical assessment and limited ability to communicate symptoms.

Center-specific age-related criteria may further contribute to these patterns. Trauma Centers 3 and 4 assigned points for children younger than 6 years, whereas Trauma Centers 1 and 2 defined pediatric triggers for children younger than 2 years and included fall criteria relative to the child’s height. Such differences in age-based criteria may increase the likelihood of younger children triggering TTA at TC3 and TC4 compared with the other centers.

### Implications for pediatric-specific TTA criteria

The mechanisms underlying pediatric overtriage remain incompletely understood and likely reflect the combined effects of prehospital visitation practices, in-hospital TTA criteria, protocol adherence, and clinician caution. Centers using point-based triage models demonstrated higher overtriage, suggesting a potential association, similar to findings in adult trauma systems but more pronounced in pediatric populations [35]. However, these findings likely reflect center-level organizational differences rather than the triage model alone.

Incorporating pediatric-specific elements, particularly age-adjusted physiological parameters, may improve triage decision-making. In the present study, systolic blood pressure was the only physiological variable available and contributed limited discriminatory value. However, prior evidence suggests that empirically derived, age-adjusted parameters such as heart rate and respiratory rate may enhance pediatric triage performance [36]. A GCS score < 13 remained a robust indicator of injury severity, consistent with findings from adult trauma populations.

Overall, the absence of pediatric-specific TTA criteria and the observed heterogeneity in organizational models suggest that greater standardization may improve equity and resource utilization while reducing unnecessary overtriage.

Several limitations should be considered when interpreting these findings. First, the cohort included only injured children transported to a level I trauma center, precluding assessment of undertriage among children managed exclusively at regional hospitals or not receiving TTA. Second, although the overall cohort was large, injury severity data were incomplete; only 44% of patients had ISS recorded. This limits representativeness and comparability, particularly relative to adult trauma cohorts in Denmark, where registry completeness is higher [21]. Patients with and without available ISS were similar in median age, transfer status, and 30-day mortality. However, ISS completeness varied between centers, ranging from 34% to 52%. Because overtriage was defined using ISS, estimates were based on the subset of patients with recorded injury severity and may therefore be subject to selection bias related to center-level differences in registry completeness. The direction of this bias is uncertain, but differential completeness across centers may lead to either over- or underestimation of overtriage rates.

In the current dataset, it was not possible to distinguish between missing AIS data and true absence of injury, which further limits the interpretation of injury severity and overtriage estimates. To our knowledge, a variable assigning an AIS exclusion code to patients receiving TTA without documented injuries has recently been introduced in the Danish Trauma Registry. Although not available in the present dataset, this variable may help improve completeness of injury severity data in future studies.

Third, the retrospective design entails inherent risks of bias, including residual confounding and limited control over data quality and completeness. Finally, the extended 11-year study period may introduce heterogeneity, as prehospital practices, TTA criteria, and organizational structures are likely to have evolved over time. Although temporal analyses indicated relatively stable overtriage rates across time intervals, such changes were not systematically captured and may still have influenced the pooled estimates. Due to incomplete injury severity data in the early study period, temporal analyses were restricted to later intervals. Nevertheless, inclusion of all four Danish level I trauma centers over a prolonged period provides a broad overview of pediatric trauma triage practices at a national level.

## Conclusion

Pediatric trauma triage in Denmark varies substantially across regions and level I trauma centers. Although prehospital visitation practices and in-hospital TTA criteria differ, all centers demonstrated consistently high rates of TTA among children with minor injuries, indicating a pattern of systemic overtriage. Together, these findings suggest a misalignment between current triage practices and the injury severity profile of pediatric trauma patients, as trauma teams are frequently activated for children with minor injuries. Greater alignment of pediatric-specific triage criteria, informed by both prehospital and in-hospital processes, may support more accurate TTA, promote consistent care, and improve resource utilization across trauma centers.

## Electronic Supplementary Material

Below is the link to the electronic supplementary material.

Supplementary Material 1

Supplementary Material 2

## Data Availability

The data that support the findings of this study are available from the corresponding authors, CHN and NR, upon reasonable request and subject to approval by the relevant Danish authorities. Due to Danish legislation and the General Data Protection Regulation (GDPR), individual-level data from national health registries cannot be made publicly available.

## Abbreviations

ED: Emergency department
EMS: Emergency Medical Service
TTA: Trauma team activation
ISS: Injury Severity Score
AIS: Abbreviated Injury Scale
GCS: Glasgow coma scale
MOI: Mechanism of injury
SBP: Systolic blood pressure
TC1-TC4: Trauma Center 1–4
