# Supplementary material for: Trauma team activation for pediatric patients in Denmark: a multicenter study of criteria, organization, and injury severity
Source: Eur J Trauma Emerg Surg. 2026 May 11;52(1):163. doi: 10.1007/s00068-026-03208-2 (PMC13160953; doi:10.1007/s00068-026-03208-2)
Supplement: Supplementary file 1 — Supplementary Material 1 [file 68_2026_3208_MOESM1_ESM.docx]

**Trauma Team Activation for Pediatric Patients in Denmark: A Multicenter Study of Criteria, Organization, and Injury Severity**

Running title: “National Variation in Pediatric Trauma Triage”

**Journal name:** European Journal of Trauma and Emergency Surgery

**Author names and affiliation:**

Christina Højfeldt Nordestgaard^1,3^, Martin Faurholdt Gude^2,3^, Sara Viskum Leth^1,3^, Nikolaj Raaber^1,3,4^

1. Research Center for Emergency Medicine, Aarhus University Hospital, Aarhus, Denmark
2. Department of Research & Development, Prehospital Emergency Medical Services, Central Denmark Region, Denmark
3. Department of Clinical Medicine, Aarhus University, Aarhus, Denmark
4. Department of Emergency Medicine, Aarhus University Hospital, Aarhus, Denmark

# **E-mail address of the corresponding author:** chrnod@rm.dk

# **Appendix 2**

**Supplementary 1:**

**Table 1:** Prehospital Visitation Criteria across Danish Regions

| **Region** | **Visitation Criteria** |
| --- | --- |
| **Region 1** | No formal prehospital criteria. One specific area, all trauma patients are transported to Trauma Center 1. In other areas of the region, transport decisions are made by the prehospital physician, particularly for severely injured patients or children. |
| **Region 2** | Predefined criteria integrated into the trauma flowchart used at trauma center 2. If no physician is present, visitation is made by EMDC or prehospital physician. |
| **Region 3** | Predefined prehospital criteria. Patients are transported to the nearest ED, except for patients meeting predefined ABCDE criteria (Supplementary 2), who are transported directly to Trauma Center 3. |
| **Region 4** | Predefined prehospital criteria. Patients are transported to the nearest ED, except for patients meeting predefined ABCDE criteria (Supplementary 2), who are transported directly to Trauma Center 4. |
| **Region 5** | Predefined prehospital criteria. Critical or unstable patients are transported to Trauma Centers 1 or 2, as decided by EMDC or prehospital physician. Other patients are transported to the nearest ED. |

Table 1 legend: EMDC = Emergency Medical Dispatch Centre; ED = Emergency Department.

**Supplementary 2:**

**Table 2:** Predefined ABCDE criteria in region 3 and 4 of Denmark:

| A: Airway | B: Breathing | C: Circulation | D: Disibillity | E: Exposure | Other |
| --- | --- | --- | --- | --- | --- |
| Unsecured airway that cannot be managed pre-hospitally | Thorax Injury  Thoracic trauma requiring thoracic surgery | Internal Bleeding / Circulatory Instability | Head Injury  Head trauma,  GCS ≤13, or unconsciousness for ≥5 minutes  Spinal trauma, Suspected transverse lesion | Burns covering >15% in adults or >10% in children  (TC2) | Amputation / Limb Reimplantation  Amputation with possible reimplantation:  (TC1)  Post-traumatic cardiac arrest  Complex injuries  (Call EMDC) |

Table 2 (legend): GCS = Glascow Coma Scale; TC1-TC2 = Trauma Center; EMDC = Emergency Medical Dispatch Centre

**Supplementary 3:**

**Table 3:** Organization of trauma team activation across level I trauma centers in Denmark

|  | **TC1** | **TC2** | **TC3** | **TC4** |
| --- | --- | --- | --- | --- |
| **Initiation of Trauma Team Activation (TTA)** | Three initiation routes:  1) Prehospital physician  2) EMS staff based on trauma criteria  3) In-ED initiation by ED physician or by protocol.  Always coordinated by inflow ED nurse | Initiated by EMS staff.  Final decision made by prehospital or EMDC physician.  A dedicated trauma secretary is contacted, who consults the trauma team leader if  in doubt. | Initiated by the prehospital physician  or EMS staff via the coordinating nurse at the trauma center.  The nurse may consult a physician in case of disagreement. | Three initiation routes:  1) Prehospital physician  2) EMS staff reports to ED physician who decides  3) In-ED initiation by ED physician or by protocol |
| **Primary Initial Trauma Team (PIT)** | **Doctors:**   - Anesthesiology specialist (team leader) and resident - Orthopedic resident - GI surgery specialist - Radiology specialist | **Doctors:**   - Anesthesiology specialist and resident - Orthopedic specialist | **Doctors:**   - Orthopedic specialist (team leader) - Anesthesiology specialist - Radiology resident | **Doctors:**   - Orthopedic specialist (team leader) - Anesthesiology specialist and resident - Emergency physician |
|  | **Nurses:**   - Anesthesiology nurse - Trauma nurses (2) | **Nurses:**   - Anesthesiology nurse - Trauma nurses (3) | **Nurses:**   - Anesthesiology nurses (2) - Trauma nurse | **Nurses:**   - Anesthesiology nurses (2) - Trauma nurse |
|  | **Other staff:**   - Bioanalyst - Specialist radiographer - Hospital service assistant - Secretary (ED) | **Other staff:**   - Bioanalysts - Radiographers (2) - Trauma service assistants (2) - Trauma secretary | **Other staff:**   - Bioanalyst - Radiographers (2) - Secretary | **Other staff:**   - Bioanalyst - Radiographer - Hospital service assistant - Secretary |
| **Pediatric Trauma Team** | TTA involving children and adolescents (0-18 years):   - Pediatrician | Newborns <4 weeks:   - Neonatologist   Children <2 years, or 2 – <10 years (if anesthetic need or comorbidities)   - Pediatric anesthesiologist   All <18 years:  Pediatrician | TTA involving children and adolescents (0-18 years):   - Pediatric anesthesiologist | TTA involving children <13 years:   - Pediatric anesthesiologist - Pediatrician - Pediatric nurse (in some cases) |
| **Total attendance** | 12 personnel | 14–18 personnel | 12 personnel | 14–16 personnel |

Table 3 (legend): TC = Trauma Center; TTA = Trauma Team Activation; EMDC = Emergency Medical Dispatch Centre; ED = Emergency Department; GI = Gastrointestinal; PIT = Primary Initial Trauma Team.

**Supplementary 4:**

**Table 4:** Trauma team activation criteria across level I trauma centers in Denmark

| Category | TC1 | TC2 | | | TC3 and TC4 |
| --- | --- | --- | --- | --- | --- |
| Structure of TTA Protocol | Checklist. TTA triggered if ≥1 criterion is met. | Flow diagram: assess physiology → anatomy → mechanism. TTA triggered if ≥1 criterion is met. | | | Point-based system. TTA triggered at ≥2 points. |
| Trauma Team Activation Criteria - Anatomical Criteria | | | | | |
| Skull / Spine / Head | Skull fracture | Paralysis after trauma; unconscious after head injury | | | Spine/neck: Paralysis (2p), Sensory disturbance (1p), fracture suspicion |
| Thorax | Flail chest | Flail chest | | | 0: No pain; 1: Tenderness; 2: Open lesion |
| Abdomen | nil | nil | | | 0: No pain; 1: Tender; 2: Open lesion |
| Fractures | ≥2 major fractures; Pelvic fx w/ instability | ≥2 long bone fx; Pelvic fracture | | | 0: None; 1: Long bone fx; 2: ≥2 or open long bone fx |
| Amputations | nil | nil | | | Amputation above hand/foot |
| TTA Criteria - Physiological Criteria (ABCD) | | | | | |
| Breathing | RR <10 or >29 | Dyspnea, bradypnea, tachypnea | | | 0: Normal; 1: Dyspnea; 2: SatO₂<90%, RR<10 or >30 |
| Circulation | Massive bleeding; SBP <100; TTA if stable patient deteriorates | SBP <90 | | | 0: SBP >90; 1: Cold/pulse >100/sweating; 2: SBP <90 |
| Disability | GCS <13 | GCS <13; unconscious after head trauma | | | 0: GCS >14; 1: GCS 13–14; 2: GCS <13 |
| TTA Criteria - Mechanism of Injury | | | | | |
| Traffic injuries | Ejection; Co-passenger dead; Trapped >30 min; Pedestrian hit | High-speed >65 km/h; ejection; motorbike >50 km/h; pedestrian hit; co-passenger dead | 0: Low energy; 1: High energy, ejection, co-passenger dead, deformity, trapped, pedestrian/cyclist | | |
| Fall accidents | Fall ≥6 meters | Fall ≥2 floors (~4m) | Fall ≥6 meters; surface noted | | |
| Penetrating trauma | Head, spine, chest, abdomen, proximal to elbow/knee | Head, spine, proximal to elbow/knee | 2 points: Head/neck | | |
| Burns | nil | Burns >15% (adult) | 2 points: Burns >15% (adult) | | |
| Other | Massive blunt trauma; Hypothermia (<32°C) | nil | nil | | |
| TTA Criteria - Specific Criteria for Children | | | | | |
| Age | <2 years | <2 years; 2– <10 years if comorbid or anesthesia need | 1 point: <6 years | | |
| Burns (pediatric) | — | Burns >10% | 2 points: Burns >10% | | |
| Falls (pediatric) | Fall ≥3 m or 2–3× height | Fall ≥3 × height | — | | |
| TTA Criteria - Increased Risk Factors | | | | | |
| Risk Groups | Children and elderly considered more vulnerable | Age <15 or >65; severe comorbidity; anticoagulation; pregnancy >20 weeks; intoxication | | 1 additional TTA point:   - Age <6 or >75 years - Comorbidity - Anticoagulant therapy - Spinal injury | |

Table 4 (Legend): TC = Trauma Center; TTA = Trauma Team Activation; GCS = Glasgow Coma Scale; SBP = Systolic Blood Pressure; RR = Respiratory Rate

**Supplementary 5:**

**Table 5:** Overtriage rates by trauma center and time interval

| Trauma Center | 2017-2019 | 2020-2022 | 2023-2024 |
| --- | --- | --- | --- |
| Trauma Center 1, % (CI) | 84.3 (71.6–92.0) | 80.1 (73.5–85.4) | 81.3 (69.8–89.0) |
| Trauma Center 2, % (CI) | 78.9 (68.3–86.7) | 81.1 (76.9–84.7) | 83.6 (78.0–88.0) |
| Trauma Center 3, % (CI) | 90.5 (77.5-96.4) | 88.9 (81.4-93.6) | 88.2 (78.2–94.0) |
| Trauma Center 4, % (CI) | NA | 95.3 (91.6–97.4) | 95.0 (88.6–97.9) |

Table 5 (Legend): NA = not applicable. Missing data was excluded from calculations. Overtriage was defined based on Injury Severity Score (ISS) thresholds as specified in the main manuscript. ISS data were not available for Trauma Center 4 in the period 2017–2019, and overtriage could therefore not be calculated for this interval. Cases with missing ISS data were excluded from the analyses.

**Supplementary 6:**

**Table 6:** Maximum abbreviated injury scale by injury severity score

| ISS | ISS 1-8  (minor) | ISS 9-15 (moderate) | ISS 16-24  (severe) | ISS ≥ 25  (Very severe) | Total |
| --- | --- | --- | --- | --- | --- |
| Maximum Abbreviated Injury Scale in each body region, N (%) | | | | | |
| Head | 304 (33) | 133 (36) | 49 (39) | 40 (44) | 526 (35) |
| Face | 133 (14) | 7 (2) | 0 (0) | 0 (0) | 140 (9) |
| Neck | 22 (2) | 3 (1) | 0 (0) | 1 (1) | 26 (2) |
| Spine | 42 (5) | 19 (5) | 9 (7) | 2 (2) | 72 (5) |
| Thorax | 76 (8) | 67 (18) | 29 (23) | 18 (20) | 190 (13) |
| Abdomen | 65 (7) | 57 (16) | 27 (22) | 19 (21) | 168 (11) |
| Extremity | 162 (18) | 77 (21) | 10 (8) | 10 (11) | 259 (17) |
| Unspecified | 121 (13) | 2 (1) | 1 (1) | 2 (2) | 126 (8) |

Table 6 (Legend): ISS = Injury Severity Score; N = Number
