# Supplementary material for: Trauma team activation for pediatric patients in Denmark: a multicenter study of criteria, organization, and injury severity
Source: Eur J Trauma Emerg Surg. 2026 May 11;52(1):163. doi: 10.1007/s00068-026-03208-2 (PMC13160953; doi:10.1007/s00068-026-03208-2)
Supplement: Supplementary file 2 — Supplementary Material 2 [file 68_2026_3208_MOESM2_ESM.docx]

**Trauma Team Activation for Pediatric Patients in Denmark: A Multicenter Study of Criteria, Organization, and Injury Severity**

Running title: “National Variation in Pediatric Trauma Triage”

**Journal name:** European Journal of Trauma and Emergency Surgery

**Author names and affiliation:**

Christina Højfeldt Nordestgaard^1,3^, Martin Faurholdt Gude^2,3^, Sara Viskum Leth^1,3^, Nikolaj Raaber^1,3,4^

1. Research Center for Emergency Medicine, Aarhus University Hospital, Aarhus, Denmark
2. Department of Research & Development, Prehospital Emergency Medical Services, Central Denmark Region, Denmark
3. Department of Clinical Medicine, Aarhus University, Aarhus, Denmark
4. Department of Emergency Medicine, Aarhus University Hospital, Aarhus, Denmark

# **E-mail address of the corresponding author:** chrnod@rm.dk

# **Appendix 1**

## **Supplementary 1**

STROBE checklist.

## **Supplementary 2**

Questions to the leading physician at trauma centers:

- **TTA criteria:** What are the trauma activation criteria for children?
- **Organization:** How is trauma activation managed organizationally (who initiates it and how)?
- **Team:** Who is included in the trauma team?
- Have there been significant changes in these aspects over the past 10 years? If possible,

describe the timing of the changes and what they entailed.
